# Prevalence of joint pain in Germany

**DOI:** 10.17886/RKI-GBE-2017-068

**Published:** 2017-10-09

**Authors:** Judith Fuchs, Franziska Prütz

**Affiliations:** Robert Koch Institute, Department of Epidemiology and Health Monitoring, Berlin

**Keywords:** JOINT PAIN, OSTEOARTHRITIS, ADULTS, HEALTH MONITORING, GERMANY

## Abstract

Joint symptoms and pain belong to the most common diseases worldwide that affect people in their usual activities and lead to loss of quality of life. 29.3% of women and 24.4% of men reported acute joint pain, which is defined as pain suffered during the past 24 hours. With age, these figures increase. Women report pain in 3.9 joints on average and men in 3.6. In both genders the joints most affected are the shoulders, knees and hips. Respondents suffering from joint pain significantly more often report medically diagnosed osteoarthritis or rheumatoid arthritis than respondents who were free of pain.

## Introduction

Joint symptoms and pain belong to the most common diseases worldwide. They affect people in their everyday lives and can lead to loss of quality of life [1]. Most commonly joint pain is caused by a musculoskeletal disease such as osteoarthritis (see the Fact sheet in this issue) or rheumatoid arthritis (RA) [2]. The diagnosis of joint diseases and provision of corresponding care leads to considerable costs to the healthcare system [3].

The International Association for the Study of Pain (IASP) defines pain as ‘an unpleasant sensory and emotional experience associated with actual or potential tissue damage, or described in terms of such damage’ [4]. There are two types of pain: acute and chronic. Acute pain lasts seconds to maximally weeks and has a clearly defined trigger. Chronic pain, in contrast, is not necessarily linked to such damaging factors and is characterised as being permanent or recurrent and lasting for a period of at least three months [5]. Chronic pain is a complex and multidimensional phenomenon influenced by somatic, psychological and social factors [6].

## Indicator

For the German Health Interview and Examination Survey for Adults (DEGS1) respondents received a questionnaire asking: ‘Have you had joint pains during the past 12 months?’ and, if yes: ‘Have you had joint pain today?’, which was more narrowly defined by adding: ‘Meaning during the past 24 hours’. This was followed by a table where participants were asked to mark the intensity of their pain during the past 24 hours. The table listed the right and left shoulders, elbows, wrists, fingers, hips, knees, ankles and toes. Pain could be marked as low, medium or strong.

The following sections present data on joint pain by gender, age and educational level, describe which joints are most commonly affected and analyse the rate at which people with joint pains are affected by medically diagnosed musculoskeletal diseases such as osteoarthritis, RA or osteoporosis. Differences between groups are interpreted as statistically significant if the respective 95% confidence intervals (CI) of relative frequencies do not overlap.

**DEGS1**

**Data holder:** Robert Koch Institute

**Objectives:** To provide reliable information about the population’s health status, health-related behaviour and health care in Germany including analysis of temporal developments and trends.

**Survey method:** Questionnaires, physical examinations and tests, a physician interview, a medication interview and laboratory investigations (blood and urine sample).

**Population:** German resident population, aged 18 and above

**Sampling:** Registry office sample; randomly selected individuals from 180 communities in Germany were invited to participate (120 original sample points of the German National Health Interview and Examination Survey 1998 and 60 new sample points).

**Participants:** N=8,151 (4,283 women; 3,868 men). The sample included persons who were newly recruited and those who had already participated in the German National Health Interview and Examination Survey 1998 (mixed design).

**Response rate:** 62% among revisiting participants and 42% first time participants

**Survey period:** 2008 to 2011

**Data protection:** This study was undertaken in strict accordance with the data protection regulations set out in the German Federal Data Protection Act and was approved by the German Federal Commissioner for Data Protection and Freedom of Information. DEGS1 was approved by the ethics committee of the Charité-Universitätsmedizin Berlin (No. EA2/047/08). Participation in the study was voluntary. The participants were fully informed about the study’s aims and content, and about data protection. All participants provided written informed consent.

More information in German is available at

www.degs-studie.de

The analyses are based on data from 7,727 participants aged between 18 and 79 (4,061 women, 3,666 men) with valid data on joint pain. The calculations were carried out using a weighting factor that corrects for deviations within the sample from the German population (as of 31 December 2010) with regard to gender, age, region and nationality, as well as district type and educational level [7]. The district type accounts for the degree of urbanisation and reflects the regional distribution in Germany. The International Standard Classification of Education (ISCED) was used to classify the responses provided on educational level [8]. Scheidt-Nave et al. contains a detailed discussion of the methodology applied in DEGS1 [9].

## Results and discussion

57.9% of women and 52.2% of men reported experiencing joint pain during the past 12 months. 29.3% of women and 24.4% of men stated acute joint pain, i.e. that they had suffered from pain during the past 24 hours. With increasing age, the percentage of people experiencing acute joint pain increases. For women it rises from 9.0% among 18- to 29-year-olds to 48% among 65- to 79-year-olds and for men from 11.4% to 34.9% respectively (Table 1).

Women with low education report joint pains more frequently (35.3%) than women from medium (28.6%) or high (24.3%) education backgrounds. No such difference exists for men.

**Figure 1:**
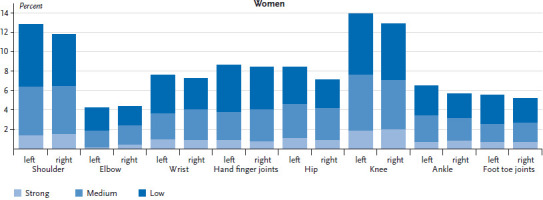
Localisation of joint pain and pain intensity among women (n=4,061) Source: DEGS1

**Figure 2:**
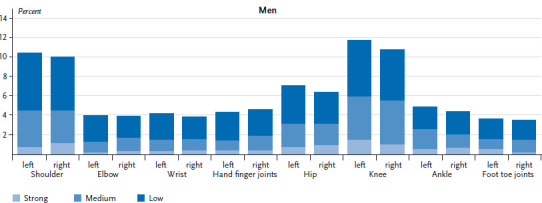
Localisation of joint pain and pain intensity among men (n=3,666) Source: DEGS1

Whilst women report that 3.9 joints were affected on average during the past 24 hours, the corresponding figure for men is 3.6. For both sexes the most frequently reported joints causing pain are the shoulders, knees and hips. Overall, 17.3% of women and 15.1% of men report knee pain, 14.1% of women and 11.6% of men shoulder pain, and 13.3% of women and 11.9% of men pain in the hip joints. Both sides of the body are equally affected (Figure 1 and Figure 2). The percentage of people experiencing strong joint pain is low, and, independently of the joint affected, strong pain is only reported by between 0.2% and 2.2% of respondents.

DEGS1 prevalence rates are slightly lower than those reported in the survey conducted in the town of Herne in 2005 [10], which conservatively estimated that 28.9% of respondents were suffering from acute joint pain. 18.2% of participants reported knee pain and 9.1% hip pain. However, the age span of respondents in this survey (40- to 95-years old) is not immediately comparable to the DEGS sample.

Women with joint pain significantly more often report a medically diagnosed osteoarthritis (46.1%, CI 42.9-49.3) than women without joint pain (12.0%, CI 10.6-15.5). For men, the corresponding share is 40.6% (CI 36.8-44.4) and 10.4% (CI 9.2-11.8). People with joint pain also reported a medically diagnosed RA more often (women: 5.7%, CI 4.4-7.4; men: 5.5%, CI 3.9-7.8) than people who were free of joint pain (women: 1.7%, CI 1.2-2.4; men: 0.7%, CI 0.4-1.3).

Further diseases such as gout, morbus Bechterew or psoriatic arthritis may also lead to joint pain. The same is true for sports or other accidents, such as in the case of a torn meniscus.

For musculoskeletal diseases, which are a major cause of joint pain, there is currently no cure. This highlights the importance of identifying those factors that effectively prevent pain and loss of functionality. Obesity and a lack of physical exercise can contribute to pain particularly in the joints of the lower extremities [11, 12]. Focus should therefore be put on preventing overweight, promoting physical exercise and the proper prescription of medicines including pain relievers.

## Key statements

- Around 29% of the women and 24% of the men surveyed reported having experienced joint pains during the past 24 hours.
- Joint pain rates increase significantly with age.
- Pain generally affects more than just one joint.
- People who report joint pain more often have osteoarthritis and rheumatoid arthritis than people without pain.

## Figures and Tables

**Table 1:** Joint pain according to gender, age and educational level (n = 4,061 women, 3,666 men) Source: DEGS1

Women	%	(95% CI)	Men	%	(95% CI)
**12-month prevalence of joint symptoms**			**12-month prevalence of joint symptoms**		
**Women**	**57.9**	**(56.1-59.6)**	**Men**	**52.2**	**(50.1-54.3)**
**Total (women and men)**	**55.1**	**(53.6-56.5)**	**Total (women and men)**	**55.1**	**(53.6-56.5)**
**Joint symptoms during the past 24 hours**			**Joint symptoms during the past 24 hours**		
**Women total**	**29.3**	**(27.7-31.0)**	**Men total**	**24.4**	**(22.9-26.1)**
**Age**			**Age**		
18-29 Years	9.0	(6.6-12.0)	18-29 Years	11.4	(8.6-14.9)
30-44 Years	16.8	(13.9-20.1)	30-44 Years	15.4	(12.7-18.6)
45-64 Years	37.9	(34.9-41.0)	45-64 Years	32.7	(30.1-35.5)
65-79 Years	48.0	(43.9-52.0)	65-79 Years	34.9	(31.1-38.9)
**Educational level**			**Educational level**		
Low education	35.3	(31.6-39.1)	Low education	22.2	(17.5-27.7)
Medium education	28.6	(26.4-30.8)	Medium education	25.7	(23.5-28.1)
High education	24.3	(21.4-27.4)	High education	23.2	(20.6-26.0)
**Total (women and men)**	**26.9**	**(25.7-28.1)**	**Total (women and men)**	**26.9**	**(25.7-28.1)**

CI=Confidence interval
